# Pre-retrieval event-related potentials predict source memory during task switching

**DOI:** 10.1016/j.neuroimage.2019.03.038

**Published:** 2019-07-01

**Authors:** Lisa H. Evans, Jane E. Herron

**Affiliations:** Cardiff University Brain Research Imaging Centre (CUBRIC), School of Psychology, Cardiff University, Cardiff, CF24 4HQ, Wales, UK

**Keywords:** Memory, Event-related potential, Pre-retrieval, Retrieval orientation, Task switching

## Abstract

Neural activity preceding memory probes differs according to retrieval goals. These divergences have been linked to retrieval orientations, which are content-specific memory states that bias retrieval towards specific contents. Here, participants were cued to retrieve either spatial location or encoding operations. On the first trial of each memory task (‘switch’ trials), preparatory ERPs preceding correct source memory judgments differed according to retrieval goal, but this effect was absent preceding memory errors. Initiating appropriate retrieval orientations therefore predicted criterial recollection. Preparatory ERPs on the second trial of each memory task (i.e. ‘stay’ trials) also differed according to retrieval goal, but the polarity of this effect was reversed from that observed on switch trials and the effect did not predict memory accuracy. This was interpreted as a correlate of retrieval orientation maintenance, with initiation and maintenance forming dissociable components of these goal-directed memory states. More generally, these findings highlight the importance of pre-retrieval processes in episodic memory.

## Highlights

• Pre-retrieval ERPs diverged according to cues signalling two different episodic tasks.
• This index predicted memory accuracy on the first trial of each task.
• The initiation of task-specific retrieval orientations influences criterial recollection.
• An orientation effect of reversed polarity on subsequent trials was linked with maintenance.
• All pre-retrieval ERP effects were maximal at frontal electrode sites.

## Graphical abstract

**Figure:**
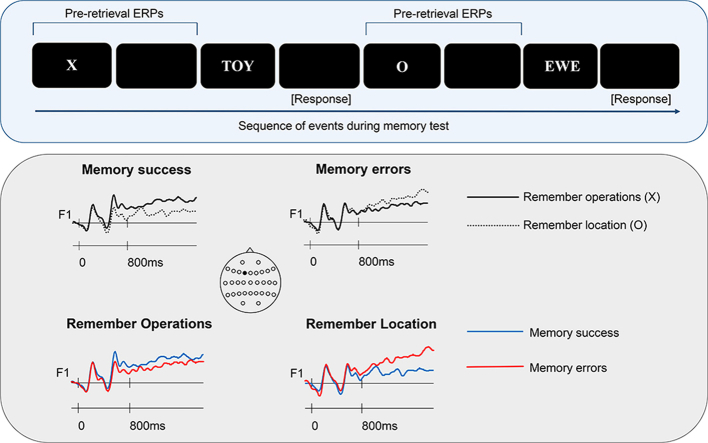

## 1 Introduction

The retrieval of information from episodic memory is guided by control processes, including those that operate prior to retrieval, preparing us to search our memories for specific kinds of information. Pre-retrieval has been characterised as an ‘early selection’ memory retrieval strategy (Jacoby, 1999), in which the retrieval of relevant mnemonic information is prioritised, and irrelevant memories are inhibited via an executive control filter (Wilckens et al., 2012). It is argued that pre-retrieval processes are dependent on the integrity of prefrontal cortex, and that both are impaired during aging (Wilckens et al., 2012). Stevens and Grady (2007) described pre-retrieval processes as controlled neurocognitive functions that support retrieval attempts, stating that a subset of these, ‘retrieval orientations’, are memory states that facilitate the retrieval of task-relevant information by influencing retrieval cue processing (Rugg and Wilding, 2000; Wilding and Ranganath, 2011). Stevens and Grady noted that this concept of retrieval orientations aligned with the proposal that dorsolateral prefrontal cortex plays a role in setting retrieval goals (Moscovitch and Winocur, 2002). Cognitive theories of memory have operationalised pre-retrieval processes as ‘descriptors’ (Burgess and Shallice, 1996) or ‘cue bias’ mechanisms (Anderson and Bjork, 1994) that shape the nature of the memory search. Despite featuring widely in memory theory, the role of pre-retrieval in episodic memory has attracted remarkably little empirical attention. Thus, in stark contrast to the large body of work identifying neural activity both before and during encoding that predicts memory (Koen et al., 2018; de Chastelaine and Rugg, 2015; Gruber and Otten, 2010), we know very little about pre-retrieval.

Brain imaging techniques have permitted neural activity to be contrasted across different retrieval tasks (Hornberger et al., 2006a; McDuff et al., 2009; Morcom and Rugg, 2012; Woodruff et al., 2006), and regions of left ventrolateral prefrontal cortex have been implicated in controlled strategic searches of memory (Badre and Wagner, 2007). However, the low temporal resolution of the hemodynamic response is not conducive to unambiguously dissociating pre-retrieval from retrieval-related neural activity (but see Polyn et al., 2005; Simons et al., 2005). Real-time techniques such as electroencephalography (EEG) have provided more temporally constrained insights into the goal-directed processing of memory probes (Bridger et al., 2009; Dzulkifli and Wilding, 2005; Hornberger et al., 2004, 2006b; Johnson and McGhee, 2015; Rosburg et al., 2011, 2013; Stenberg et al., 2006; Werkle-Bergner et al., 2005) and goal-related neural activity preceding memory probes. With regard to the latter, a number of studies focused upon neural differences between preparing to make episodic or non-episodic judgments, and it was repeatedly observed that preparing to remember episodic information elicits enhanced slow-wave activity over right frontal scalp sites (Duzel et al., 1999, 2001; Evans et al., 2015; Herron and Wilding, 2004, 2006a; Morcom and Rugg, 2002). This effect has been linked to ‘retrieval mode’, a memory state invoked whenever episodic retrieval is required and which remains invariant across different episodic retrieval requirements (Tulving, 1983).

A further series of studies reported additional divergences between event-related potentials (ERPs) time-locked to pre-stimulus cues signalling different episodic tasks (Herron et al., 2016; Herron and Wilding, 2004, 2006b). These cues directed participants to prepare to retrieve either the physical spatial location (left or right of fixation) or the encoding operations associated with test items, and the associated preparatory divergences were characterised as correlates of distinct content-specific retrieval orientations. The differences were maximal at left anterior sites in two of these studies (Herron and Wilding, 2004, 2006b), consistent with fMRI studies reporting differential activation of ventrolateral and dorsolateral prefrontal cortex in accordance with episodic retrieval goals (Dobbins et al., 2003; Dobbins and Wagner, 2005; Simons et al., 2005; Woodruff et al., 2006), although inferring the neuroanatomical substrates giving rise to scalp-recorded ERPs is not straightforward. While cues signalling different episodic memory goals elicit significant differences in preparatory neural activity, these differences have not yet been linked to subsequent memory accuracy. Given that the functional role assigned to retrieval orientations is to facilitate the retrieval of task relevant information, it is important to establish whether these preparatory divergences predict source memory accuracy.

In an important development of this research, we recently employed the ‘subsequent memory’ approach (Paller et al., 1987; Paller and Wagner, 2002), in which neural activity preceding accurate memory judgments and memory errors is contrasted. We demonstrated that pre-retrieval ERPs elicited by an episodic cue (retrieve encoding operations) were more positive going than those elicited by a non-episodic cue, but that this effect was only evident prior to correct memory judgments (Herron and Evans, 2018). We also observed that frontal ERPs were more positive-going prior to correct source memory judgements than those prior to memory errors - in essence, a ‘preparatory memory effect’. These differences remained for a subgroup of participants for whom ERPs preceding correct source judgments could be contrasted with ERPs predicting correctly recognised items for which source memory failed, indicating that this preparatory index is linked to, and beneficial for, criterial recollection (Yonelinas and Jacoby, 1996). For this reason, the index was linked to retrieval orientation as opposed to retrieval mode. These divergences occurred only on the first trial of each cue-type, indicating that it was the *initiation* of the retrieval orientation that was important for memory success.

A similar ERP study recently reported by Xia et al. (2018) required younger (19–29yrs) and older (60-79ys) participants to retrieve conceptual spatial locations (e.g. ‘garden’) associated with test items (e.g. ‘doll’). These authors reported that pre-retrieval frontal ERPs predicted recollection of spatial location for both young and older adults between 1100 and 2000 ms but with an earlier onset for older adults (200 ms). This effect differed in polarity from that reported by Herron and Evans (2018), with ERPs preceding associative hits (i.e. recognised items accompanied by correct location responses) being more *negative* going than those preceding associative misses (recognised items accompanied by incorrect location responses). It is unclear whether this polarity reversal is due to methodological differences between the two studies or whether preparatory memory effects are content-specific. A further EEG study explored pre-retrieval oscillations during a source memory task which required participants to remember the encoding task associated with each test item (Addante et al., 2011). These authors reported that fronto-parietal theta predicted the successful recollection of encoding task information. The latency of this enhancement - 150–300 ms pre-stimulus - was considerably shorter in duration than the ERP preparatory memory effects reported by Xia et al. (2018) and Herron and Evans (2018). Importantly, although all three of these studies indicate a role for pre-retrieval processes in the recollection of contextual information, the lack of a second memory task means that it is difficult to determine whether they are indexing task-specific processes such as retrieval orientation or more general processes such as retrieval mode.

The present study aimed to resolve this ambiguity by examining whether preparatory ERPs associated with two source memory tasks (thus eliminating mode) predicted source memory accuracy. If retrieval orientations facilitate the retrieval of task relevant information, then neural correlates of these orientations should be larger preceding accurate memory judgments than preceding memory errors. The design of this experiment also permitted an exploration of whether within-task preparatory memory effects (i.e. neural activity differentiating subsequent source memory success from errors) are equivalent across the two memory tasks or whether they differ according to episodic content.

## 2 Method

### 2.1 Participants

Ethical approval for the study was granted by Cardiff University's School of Psychology ethics committee. Participants were drawn from the undergraduate population at Cardiff University, and participated on a voluntary basis in return for financial renumeration after giving informed consent. Data from two participants were excluded from analysis because they made too few memory errors to allow ERPs to be formed for this response category. The remaining 24 participants were all right-handed native English speakers (23 were female) with a mean age of 18.6 years (range: 18–20).

### 2.2 Design

Stimuli were 480 nouns (concreteness range = 500–700) selected from the MRC psycholinguistic database (Coltheart, 1981) with Kucera-Francis frequencies of 1–9 per million. Words were 3–9 letters long (e.g. FAWN, KENNEL, MAGICIAN, THIMBLE) and were presented in white capitalised Times New Roman font on a black background. The experiment consisted of five study-test blocks. At study, participants alternated between an animate/inanimate task and an indoors/outdoors task four times, performing the specified task until the alternate study instructions appeared. Within each task, half of items were presented to the left of fixation and half to the right. Each study list comprised 72 words with an additional 24 new words presented at test. At test each word was preceded by one of two cues (X or O) which directed participants to prepare to retrieve either the encoding operations (animacy or indoors/outdoors task) or the left/right screen location associated with the item at study. The mapping of symbol to cue type was counterbalanced across participants. Each cue type was presented for 2 consecutive trials to permit each cue type to be separated according to whether the cue was different from that on the preceding trial (*switch* trials) or the same (*stay* trials).

Each test block employed 48 operations cues and 48 location cues. The ratio of old to new items following both cues was 3:1. A reduced number of unstudied items were employed so as to permit a separation of cue data according to subsequent retrieval accuracy within a recording period of reasonable length. The old/new status of words, the mapping of X/O to task and the assignment of words to encoding operations and screen location were fully counterbalanced across participants.

### 2.3 Procedure

Each participant completed one practice study-test cycle. At study, participants performed the encoding task specified by the onscreen instruction, responding via button press with their left or right hand depending on whether the word was animate/inanimate or most likely to be found indoors/outdoors respectively. A fixation asterisk (1000 ms) preceded the study word (300 ms) then the screen remained blank until 500 ms after a response was made.

At test, Operations cues required participants to remember whether the subsequent word had been presented in the animate/inanimate task, the indoor/outdoor task, or was new. Location cues required participants to remember whether the word had been presented on the left, the right, or was new. Participants responded via button press, using the index finger of one hand for new responses and the index and middle fingers of the other hand for the remaining responses, and were encouraged to balance speed and accuracy equally. The mappings between left/right hand and response types were counterbalanced across participants. The preparatory cue (300 ms) was followed by an asterisk (2000 ms) and then the test word (300 ms). The screen then remained blank until 500 ms after a response was made.

### 2.4 Electroencephalogram (EEG) acquisition and analysis

EEG was recorded with a Biosemi ActiveTwo amplifier from 32 locations based on the International 10–20 system (Jasper, 1958). Additional electrodes were placed on the mastoid processes. EOG was recorded from above and below the left eye (VEOG) and from the outer canthi (HEOG). EEG (range DC-419 Hz; sampling rate 2048 Hz) was acquired referenced to linked electrodes located midway between POz and PO3/PO4 respectively, and was re-referenced off-line to linked mastoids. Data was bandpass filtered off-line (0.03–40 Hz) and downsampled to 125 Hz, resulting in a total epoch length of 2048 ms inclusive of a 104 ms baseline relative to which all mean amplitudes were computed. Trials containing large EOG artefact were rejected, as were trials containing A/D saturation or baseline drift exceeding ±80 μV. Artefact rejection was first implemented by an automated procedure, then verified visually with the experimenter blind to the behavioral status of each trial. Blink artefacts were corrected using a linear regression estimate (Semlitsch et al., 1986). A 7-point binomially weighted smoothing filter was applied prior to analysis. The behavioral and EEG data can be accessed on the Open Science Framework (https://osf.io/nmxv2/).

## 3 Results

### 3.1 Behavior

Table 1 shows behavior at test separated according to Retrieval Task and Switch/Stay trial status. ANOVA of correct responses incorporating the factors of Retrieval Task (Operations/Location), Response Type (Source Hit/Correct Rejection) and Switch/Stay trial status gave rise to a main effect of Response Type [*F*_(1,23)_ = 149.62, *p* < 0.001] reflecting greater accuracy for new than for studied items. No effects of Retrieval Task or Switch/Stay were detected. Additional ANOVAs of both old/new discrimination (p_hit_–p_false alarm_) and conditional source accuracy (study items attracting correct source judgements expressed as a proportion of correctly recognised items) also revealed no effects of Retrieval Task or Switch/Stay.

**Table 1:** Response accuracy and associated RTs (in ms) for the Operations and Location memory tasks on Switch and on Stay trials (standard deviations in parentheses). Source Hit = accurate source memory judgments, Hit-Miss = recognised items associated with source errors, Miss = studied items given a ‘new’ response, Correct Rejection = new items correctly given a ‘new’ response.

	**Switch trials**		**Stay trials**	
	Accuracy	RT	Accuracy	RT
***Episodic Operation******s***				
Source Hit	.58 (.13)	1963 (945)	.61 (.11)	1741 (717)
Hit-Miss	.29 (.11)	1948 (908)	.27 (.09)	1782 (759)
Miss	.13 (.08)	1296 (665)	.12 (.09)	1427 (810)
Correct Rejection	.84 (.14)	981 (447)	.86 (.12)	933 (386)
***Episodic Location***				
Source Hit	.57 (.12)	1386 (566)	.60 (.13)	1246 (497)
Hit-Miss	.30 (.06)	1573 (657)	.29 (.09)	1553 (672)
Miss	.13 (.09)	1240 (690)	.11 (.07)	1137 (605)
Correct Rejection	.85 (.11)	966 (569)	.82 (.17)	938 (382)

ANOVA of RT data associated with correct responses incorporated the factors of Retrieval Task (Operations/Location), Response Type (Source Hit, Correct Rejection) and Switch/Stay trial status. This analysis revealed main effects of Retrieval Task [*F*_(1,23)_ = 22.99, *p* < 0.001], Response Type [*F*_(1,23)_ = 61.75, *p* < 0.001], Switch/Stay [*F*_(1,23)_ = 17.27, *p* < 0.001], and interactions between Switch/Stay x Response Type [*F*_(1,23)_ = 5.99, *p* < 0.05] and Retrieval Task x Response Type [*F*_(1,23)_ = 28.42, *p* < 0.001]. These interactions arose because Source Hit responses were significantly faster in the Location (*M* = 1852, 95% CI = [1520, 2180]) than the Operations task (*M* = 1316, 95% CI = [1110, 1530], *t*_(1,23)_ = 5.33, *p* < 0.001, Cohen's d_z_ = 1.09, Hedges g_av_ = 0.78), and were also significantly faster on Stay (*M* = 1498, 95% CI = [1260, 1740]) than on Switch trials (*M* = 1688, 95% CI = [1390, 1980] *t*_(1,23)_ = 3.93, *p* = 0.001, Cohen's d_z_ = 0.80, Hedges g_av_ = 0.28] whereas Correct Rejection RTs were unaffected by either Retrieval Task or Switch/Stay.

### 3.2 Event-related potentials

Averaged ERPs were formed for each participant time-locked to the Operations and Location cues preceding memory probes, and were further separated according to whether source memory judgments to subsequent test items were correct (Source Hits) or not (Errors), and whether cues were presented on switch or stay trials. The ‘Errors’ category consisted of a weighted average of recognised items associated with incorrect source judgments and studied items that were not recognised at all. The mean numbers of trials (ranges in parentheses) contributing to each condition of interest were as follows: Operations Source Hits Switch = 46 (29–64), Operations Errors Switch = 34 (16–55), Location Source Hits Switch = 47 (21–69), Location Errors Switch = 34 (17–48), Operations Source Hits Stay = 49 (28–65), Operations Errors Stay = 32 (19–51), Location Source Hits Stay = 48 (25–74), Location Errors Stay = 31 (16–57).

Prior research using this pair of retrieval tasks identified preparatory correlates of retrieval orientation between 700 and 1900 ms post-cue (Herron and Wilding, 2006b). Mean amplitudes of averaged ERPs time-locked to cues were therefore measured during this time window at 24 sites distributed across the scalp (F1/F2, F3/F4, F5/F6, F7/F8, C1/C2, C3/C4, C5/C6, T7/T8, P1/P2, P3/P4, P5/P6, P7/P8) and subjected to a repeated measures ANOVA which included the experimental factors of Retrieval Task (Operations/Location), Accuracy (Source Hits/Errors), Switch/Stay trial status, and the electrode site factors of Anterior/Central/Posterior dimension, Hemisphere (left/right) and Site (inferior/mid-lateral/superior/midline). In line with previous research, significant effects of Switch/Stay were followed up with separate ANOVAs on switch and stay trial data. Where licensed by significant interactions between Retrieval Task and Accuracy, further subsidiary analyses tested for orientation-related effects of Retrieval Task for Source Hits and for Errors, and for preparatory memory effects of Accuracy within each retrieval task. Main effects and highest order interactions involving Retrieval Task are reported. All analyses included the Greenhouse-Geisser correction for non-sphericity where necessary (Greenhouse and Geisser, 1959). Epsilon-corrected degrees of freedom are given in the text.

**Fig. 1:**
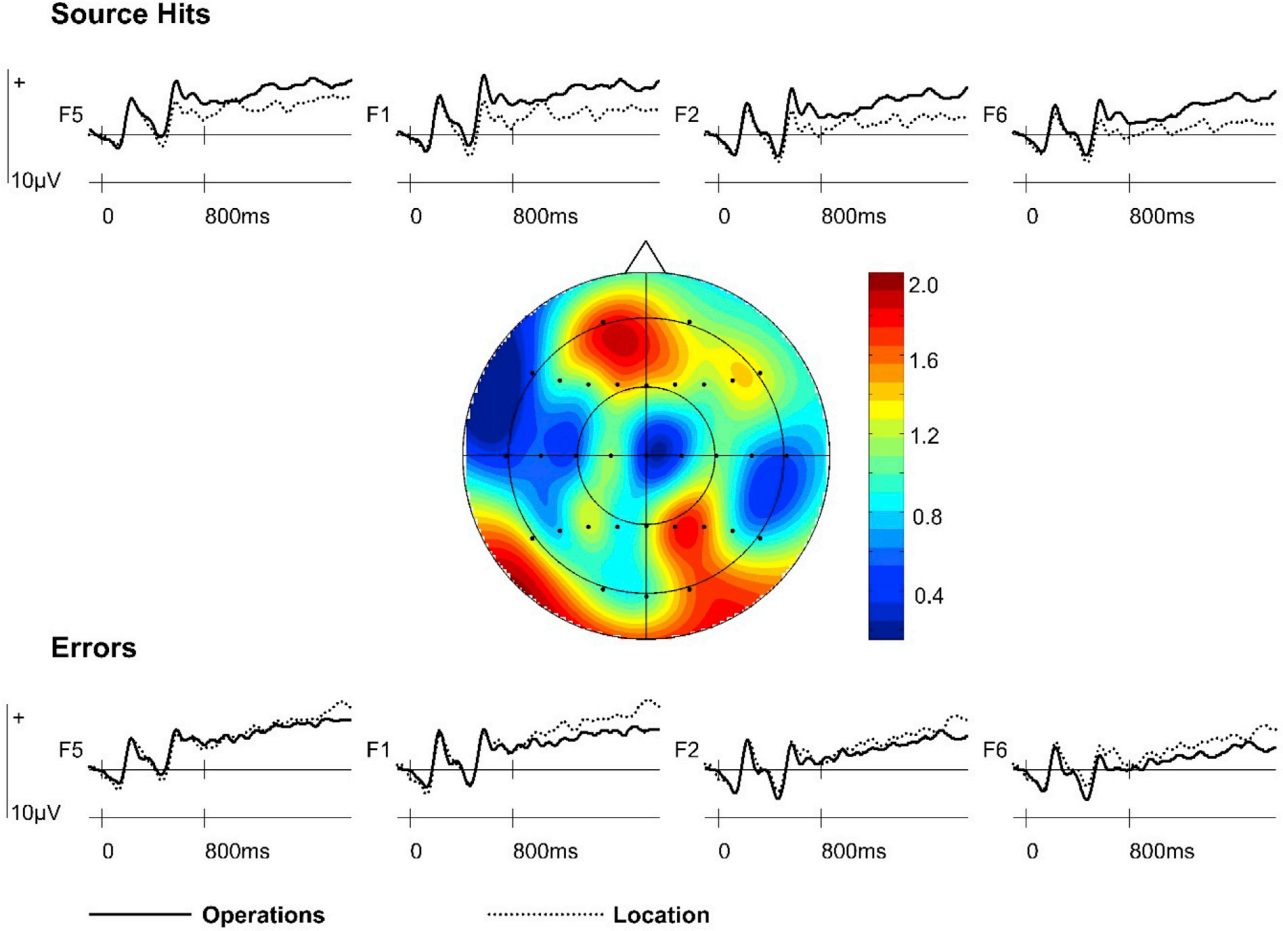
Switch trial ERPs time-locked to Operations and Location preparatory cues at bilateral frontal (F5, F1, F2, F6) electrode sites separated by subsequent response accuracy. The topographic map (nose indicates front of head) shows the scalp distribution of the significant effect of Retrieval Task observed for Source Hits between 700 and 1900 ms (data were formed by subtracting averaged ERP amplitudes associated with Location Source Hits from Operations Source Hits).

The global ANOVA revealed an interaction between Switch/Stay x Retrieval Task x Accuracy [*F*_(1,23)_ = 4.33, *p* < 0.05], Retrieval Task x Hemisphere x Site [*F*_(2.7,62.1)_ = 3.25, *p* < 0.05] and Accuracy x Anterior/Posterior x Site [*F*_(4.7,109.1)_ = 2.57, *p* < 0.05]. Analysis of switch trial ERPs revealed an interaction between Retrieval Task x Accuracy [*F*_(1,23)_ = 5.40, *p* < 0.05], reflecting greater positivity for Operations Source Hits than for Location Source Hits and a smaller effect of reversed polarity for Operations and Location Errors (see Fig. 1). A main effect of Retrieval Task was observed in the subsidiary analysis performed on Operations and Location Source Hits [*F*_(1,23)_ = 4.38, *p* < 0.05] but no effect of Retrieval Task was evident in the subsidiary analysis of Errors.

**Fig. 2:**
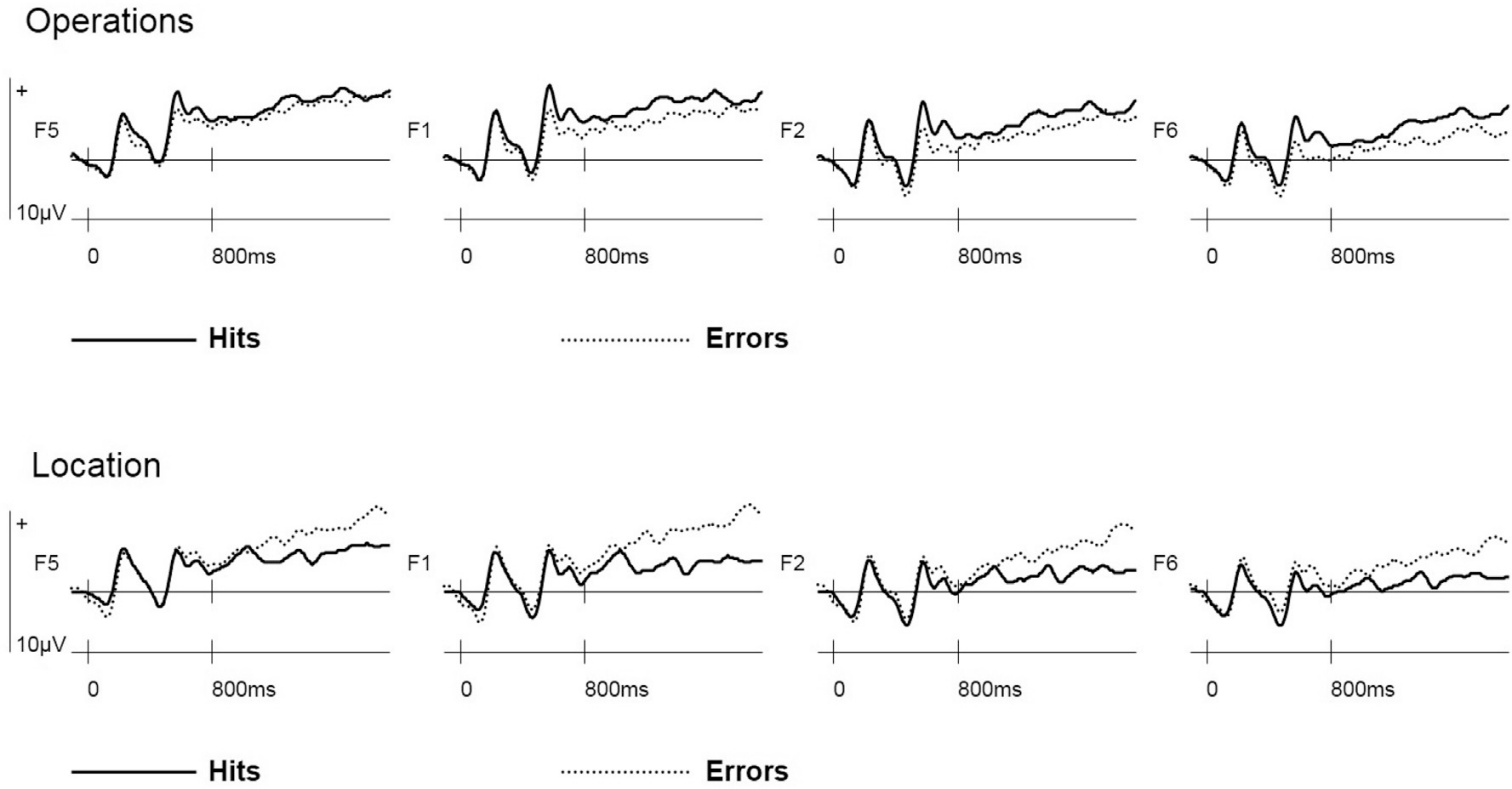
Switch trial ERPs preceding Source Hits and Errors at bilateral frontal (F5, F1, F2, F6) electrode sites separated by retrieval task (Operations, Location).

When the interaction was explored from the perspective of accuracy effects within each retrieval task, a main effect of Accuracy was found for the Location cue [*F*_(1,23)_ = 5.70, *p* < 0.05], reflecting greater negativity for Location Source Hits than for Location Errors (see Fig. 2). A smaller accuracy effect of reversed polarity visually apparent for Operations cue ERPs was not statistically significant.

**Fig. 3:**
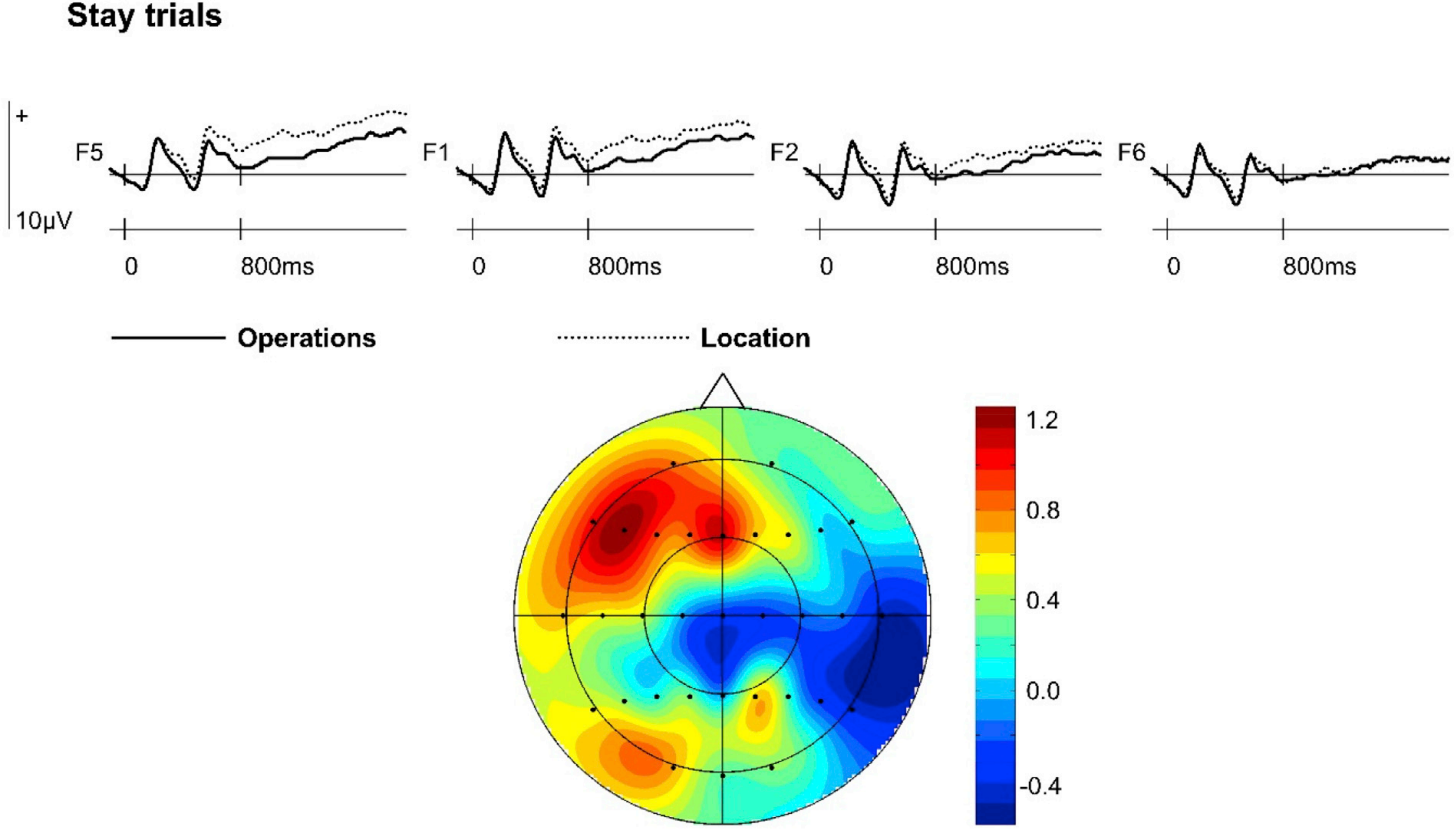
Stay trial ERPs time-locked to Operations and Location preparatory cues at bilateral frontal (F5, F1, F2, F6) electrode sites, collapsed across subsequent response accuracy. The topographic map (nose indicates front of head) shows the scalp distribution of the significant retrieval orientation effect observed on stay trials between 700 and 1900 ms (data were formed by subtracting averaged ERP amplitudes associated with Operations ERPs from Location ERPs).

Analysis of cue-related ERPs on stay trials revealed an interaction between Retrieval Task x Hemisphere x Site [*F*_(1.8,42.0)_ = 3.55, *p* < 0.05], reflecting greater positivity for Location Cues maximal at left inferior sites (see Fig. 3). A main effect of Retrieval Task was observed in a post-hoc analysis restricted to left inferior sites [*F*_(1,23)_ = 8.81, *p* < 0.05]. No effects of subsequent retrieval accuracy on stay trials were detected in these analyses.

### 3.3 Topographic analyses

Topographic analyses examined whether ERP effects of retrieval orientation that reached significance in the first stage analyses differed in their scalp distributions. Specifically, the Operations-Location effect observed for Source Hits on switch trials was contrasted with the accuracy-insensitive Operations-Location effect of opposite polarity observed on stay trials. This analysis was conducted on the difference scores obtained by subtracting Location from Operations ERPs in each of the contrasts described above from all 32 electrode sites. The data were rescaled using the max–min method to avoid confounding changes in amplitude with changes in the shape of scalp distributions (McCarthy and Wood, 1985), and the resulting ANOVA included the factors of Switch/Stay and Electrode Site. No reliable effects involving Switch/Stay were observed.

## 4 Discussion

While a large number of EEG and fMRI studies have examined the influence of different episodic memory goals on neural activity during retrieval, there has been little evidence that these measures of retrieval orientation facilitate retrieval success (but see Bridger et al., 2009 for an individual differences approach). While pre-retrieval EEG/ERPs have been linked with subsequent source memory accuracy in a small number of studies, these have all used a single memory task (Addante et al., 2011; Herron and Evans, 2018; Xia et al., 2018). Contrasting two different episodic tasks in the present study allowed the functional significance of this measure to be constrained to retrieval orientation, as general episodic processes linked to retrieval mode should be common to both memory tasks. The critical finding from the present study is that pre-retrieval measures of retrieval orientation predicted source memory accuracy, being evident prior to correct source memory judgments but not prior to memory errors. This index has previously been linked to the initiation of retrieval orientations, such as task-set configuration, as opposed to the ongoing maintenance of orientations throughout tasks. This is because it is observed on switch and not on stay trials, and is evident when participants frequently alternate between retrieval tasks but not when retrieval goals are maintained across items (Herron and Wilding, 2006b). The initiation of retrieval orientations has been linked to activation in left lateral anterior prefrontal cortex in a fMRI study using a similar design (Simons et al., 2005). Importantly, the present findings indicate that the extent to which an appropriate retrieval orientation is initiated predicts the successful retrieval of goal-relevant contextual information. This is a substantial step forward in understanding the benefits that neural measures of retrieval orientation confer at retrieval. Analysis of the behavioral data revealed statistically equivalent levels of source memory accuracy in the two retrieval tasks. Task-specific differences in ERPs preceding source hits are therefore unlikely to reflect differences in general processes such as effort or attention.

A pre-retrieval effect of orientation was also observed on stay trials, but this was not moderated by subsequent memory accuracy. This effect was also of reversed polarity to that observed on switch trials. The topographic analysis did not indicate that the orientation effects observed on switch and on stay trials arose from different neural populations, but clearly the process(es) indexed by these effects were not equivalent. The left anterior scalp distributions of both effects are consistent with previously reported measures of retrieval orientation in ERP (Herron and Wilding, 2004, 2006b) and fMRI studies (Dobbins et al., 2003; Dobbins and Wagner, 2005; Simons et al., 2005; Woodruff et al., 2006), as well as accounts that link left dorsolateral prefrontal cortex with the setting of retrieval goals (Moscovitsch and Winocur, 2002) and left ventrolateral cortex with the controlled strategic search of memory (Badre and Wagner, 2007). Although the location of effects in ERP and fMRI study data are consistent it is important to note that a frontal scalp distribution found in ERP analyses does not necessarily imply that the source is actually in the prefrontal cortex. As participants were required to overtly switch between memory tasks in response to pre-stimulus cues, the present data are also consistent with Wilckens et al.‘s (2012) description of pre-retrieval as an early selection retrieval strategy linked to executive control and dependent on prefrontal cortex. If switch trial ERPs reflect processes involved in the initiation of retrieval orientations, then the effect observed on stay trials may reflect processes involved in the maintenance of retrieval orientations across subsequent trials. This interpretation logically follows from the conceptualisation of retrieval orientations as sustained memory states that influence the processing of subsequent stimuli (Rugg and Wilding, 2000). Evidence for this view comes from a mixed design fMRI study comparing retrieval orientations for items studied as words versus pictures, and reporting supra-item activations in medial and lateral prefrontal cortex which were sustained throughout memory tasks (Woodruff et al., 2006).

Supporting a maintenance interpretation, the stay trial ERPs observed here closely resemble those obtained in a recent experiment which maintained retrieval goals across multiple successive trials (Herron, 2018). Frontal pre-retrieval ERPs were more positive-going when participants retrieved visualisation-based information than when they retrieved encoding operations, and this was interpreted as a direct correlate of retrieval orientation maintenance across trials. The effect was eliminated in a group of participants who first completed a stroop task (implicating a role for reserves of cognitive control in the maintenance of retrieval orientations) while no corresponding decrease in memory accuracy was observed, which is consistent with the insensitivity of the stay trial effect to memory accuracy observed here. A double dissociation was also observed between ERP indices of retrieval orientation maintenance and an established measure of post-retrieval monitoring, indicating that orientations enhance retrieval efficiency. Similarly, there is some evidence in the present study that maintaining an orientation increased retrieval efficiency as reaction times associated with correct source judgments were faster on stay than on switch trials (this RT decrease was not observed for correct rejections). There have been few previous experimental opportunities to observe the transition between initiation and maintenance of retrieval orientation. In one such study, while preparatory correlates of initiation were observed on switch trials, no effects of retrieval goal were detected on stay trials (Herron and Wilding, 2006b). It is notable, however, that some of the test blocks in this study (Experiment 1b) used an unpredictable trial sequence in which retrieval goals sometimes changed after a single trial, which may have discouraged participants from maintaining retrieval orientations.

The interaction between retrieval task and memory accuracy on switch trials also indicated that preparatory memory effects were not equivalent across retrieval tasks. By separating neural activity preceding accurate and inaccurate memory judgments, preparatory memory effects have previously been demonstrated within a single source memory task using electrophysiological data (Addante et al., 2011; Xia et al., 2018; Herron and Evans, 2018). In the present study, pre-retrieval ERPs predicted source memory accuracy in the Location task, with Location Source Hits eliciting more negative-going ERPs than Location Errors. This effect closely resembles that reported by Xia et al. (2018), who also reported more negative-going ERPs at frontal sites prior to associative memory for spatial information than recognised items for which spatial information could not be recalled. Intriguingly, the effect reported by Xia et al. predicted memory for conceptual spatial information such as the word ‘GARDEN’, whereas the effect observed here predicted memory for physical screen location (i.e. left or right of fixation). These combined findings indicate that this preparatory memory effect supports a broad range of location-based memories.

In our previous study (Herron and Evans, 2018), we reported a sustained positive shift in ERPs preceding correct source judgments at frontal electrode sites when participants were required to retrieve encoding operations. A visually similar preparatory memory effect for the same retrieval task was not statistically significant here. This effect may have been attenuated for a number of reasons. The three main differences between the two studies were i) the requirement to switch between two episodic tasks rather than between an episodic and a non-episodic task, ii) the requirement to switch to Operations from a more difficult task in the present experiment than in the prior study (accuracy in alternate task = 0.60 vs 0.87 respectively), and iii) the levels of memory accuracy obtained in the Operations task were lower here (0.61) than previously (0.68). One or more of these factors may have influenced neural activity linked to preparation for the Operations task on switch trials.

The oscillatory EEG study reported by Addante et al. (2011) also demonstrated a significant role for pre-stimulus processes in the successful retrieval of encoding operations. These authors reported that pre-stimulus theta at left temporal, left parietal and mid frontal electrode sites was enhanced prior to correct source judgments when compared with recognised items associated with incorrect source judgments. They also found that larger pre-stimulus theta was related to better memory performance both across and within subjects, and that it was positively correlated with post-stimulus left parietal theta associated with recollection. The authors proposed that preparatory processes may directly impact post-stimulus retrieval processing, and that episodic retrieval ‘reflects an interaction between cues and one's preceding neurocognitive state’. A recent tDCS-EEG study (transcranial direct current stimulation) replicated the pre-retrieval effect in the sham condition, and also found that anodal tDCS (theta frequency) during the study-test interval over left dorsolateral prefrontal cortex eliminated this pre-retrieval effect and reduced source memory accuracy (Mizrak et al., 2018). Due to the temporal and topographic differences between oscillatory and ERP preparatory memory effects, it seems unlikely that they are indexing exactly the same process, but it is evident that both measures of electrophysiological activity indicate an important role for preparatory processes linked to neurocognitive states during retrieval. They also echo electrophysiological studies of memory encoding, which show that pre-stimulus neural activity at study predicts subsequent memory accuracy at test (Gruber and Otten, 2010; Guderian et al., 2009; Otten et al., 2006, 2010).

Taken in combination, data from the small number of pre-retrieval EEG studies conducted thus far indicate that preparatory memory effects are not equivalent across different retrieval tasks, but that they instead reflect variations in task-specific retrieval orientations, with pre-retrieval ERPs predicting memory accuracy in a content-specific manner. While theoretical accounts of pre-retrieval propose that prefrontal cortex is involved in setting retrieval goals and initiating memory searches, Polyn et al. (2005) reported fMRI evidence for the precise cortical implementation of task-specific memory searches during pre-retrieval. Participants studied three classes of words (celebrities, objects and landmarks) and a multivoxel pattern classification algorithm identified distinct patterns of neural activity associated with each class of item during encoding. The classifier was then used during a free recall task, and activation of category-specific patterns of neural activity were observed in the seconds before items from that category were recalled. These activations were observed in ventral temporal cortex, medial temporal cortex and prefrontal cortex. Similarly, Sederberg et al. (2007) found that intracranial EEG gamma oscillations that predicted memory during encoding also reactivated in the 500 ms prior to recollection in a free recall task, differentiating correct recall from memory errors. These oscillations were observed in regions corresponding with those identified by Polyn et al. (2005), including left hippocampal, left temporal and left prefrontal regions (see also Morton et al., 2013). Although these experiments examined self-initiated free recall as opposed to the criterial source memory tasks used here, they provide strong evidence that content-specific pre-retrieval processes guide memory retrieval by initiating memory states that correspond with those active during encoding (Polyn et al., 2005).

In conclusion, we have demonstrated that pre-retrieval ERP correlates of orientation were evident prior to memory probes eliciting correct source judgments but not prior to test items eliciting memory errors. The fact that this effect predicted memory success on switch trials suggests that the initiation of appropriate retrieval orientations influences the successful recovery of criterial contextual information. Furthermore, the present findings in conjunction with those from other studies (Xia et al., 2018; Herron and Evans, 2018) demonstrate that pre-stimulus ERPs not only predict whether source information will be recollected, but do so differentially depending on the memory contents that are to be recovered. Preparatory correlates of retrieval orientation on stay trials were i) of reversed polarity to those observed on switch trials, and ii) insensitive to subsequent memory accuracy. We propose that this effect reflects the maintenance of retrieval orientations across subsequent stimuli, and illustrates the transition from initiation to maintenance of orientations within the same experimental context for the first time. The frontal scalp distributions of these effects are consistent with the view that regions within prefrontal cortex implement task-specific memory searches during pre-retrieval, although further studies combining high density electrophysiological recordings with source localisation analyses are required to confirm this link.
